# Means, Motive, and Opportunity: Do Non-Islet-Reactive Infiltrating T Cells Contribute to Autoimmunity in Type 1 Diabetes?

**DOI:** 10.3389/fimmu.2021.683091

**Published:** 2021-06-16

**Authors:** Teresa Rodriguez-Calvo, Gustaf Christoffersson, Christine Bender, Matthias G. von Herrath, Roberto Mallone, Sally C. Kent, Eddie A. James

**Affiliations:** ^1^ Institute of Diabetes Research, Helmholtz Zentrum München, German Research Center for Environmental Health, Munich-Neuherberg, Germany; ^2^ German Center for Diabetes Research (DZD), Neuherberg, Germany; ^3^ Science for Life Laboratory, Uppsala University, Uppsala, Sweden; ^4^ Department of Medical Cell Biology, Uppsala University, Uppsala, Sweden; ^5^ Center for Autoimmunity and Inflammation, Type 1 Diabetes Center at La Jolla Institute for Immunology, La Jolla, CA, United States; ^6^ Université de Paris, Institut Cochin, CNRS, INSERM, Paris, France; ^7^ Assistance Publique Hôpitaux de Paris, Cochin Hospital, Service de Diabétologie et Immunologie Clinique, Paris, France; ^8^ Department of Medicine, Division of Diabetes, Diabetes Center of Excellence, University of Massachusetts Medical School, Worcester, MA, United States; ^9^ Translatonal Research Program, Benaroya Research Institute, Seattle WA, United States

**Keywords:** type 1 diabetes, insulitis, autoreactive T cells, non-islet reactive T cells, β cell destruction

## Abstract

In human type 1 diabetes and animal models of the disease, a diverse assortment of immune cells infiltrates the pancreatic islets. CD8^+^ T cells are well represented within infiltrates and HLA multimer staining of pancreas sections provides clear evidence that islet epitope reactive T cells are present within autoimmune lesions. These *bona fide* effectors have been a key research focus because these cells represent an intellectually attractive culprit for β cell destruction. However, T cell receptors are highly diverse in human insulitis. This suggests correspondingly broad antigen specificity, which includes a majority of T cells for which there is no evidence of islet-specific reactivity. The presence of “non-cognate” T cells in insulitis raises suspicion that their role could be beyond that of an innocent bystander. In this perspective, we consider the potential pathogenic contribution of non-islet-reactive T cells. Our intellectual framework will be that of a criminal investigation. Having arraigned islet-specific CD8^+^ T cells for the murder of pancreatic β cells, we then turn our attention to the non-target immune cells present in human insulitis and consider the possible regulatory, benign, or effector roles that they may play in disease. Considering available evidence, we overview the case that can be made that non-islet-reactive infiltrating T cells should be suspected as co-conspirators or accessories to the crime and suggest some possible routes forward for reaching a better understanding of their role in disease.

## Introduction

In criminal investigations, establishing means, motive, and opportunity are necessary elements to prove a suspect’s guilt. In this construct, means refers to available tools or methods to commit the crime; motive refers to a plausible reason for committing the crime, and opportunity is the occasion that presents itself to allow the crime to occur (1). In this perspective, we explore the role and function of non-islet-reactive immune cells in human insulitis. Type 1 diabetes (T1D) involves the criminal activity of murder of the insulin-producing pancreatic islet β cells. In this context, “means” is established by showing that a suspect possesses an adequately potent murder weapon (effector function); “motive” is established by showing that the suspect was driven by some plausible impulse to commit the crime (typically, an islet-reactive antigen receptor); and “opportunity” is demonstrated by placing the suspect at the scene of the crime and eliminating any “alibi”. There is increasing evidence suggesting that β-cell metabolic dysfunction may directly contribute to β-cell stress and demise (2), and, potentially, to the autoimmune attack (“assisted suicide”) (3, 4). Furthermore, some evidence suggests that neonatal remodeling events (waves of apoptosis) may lead to “incompetent repairmen”, leaving vulnerabilities that set the stage for the crime (revealed when the perpetrators “case the joint”) (5). However, the focus of this mini-review is to investigate the role of T cells at the scene of the crime. We will present ample evidence of opportunity: cells with no discernable evidence of islet antigen reactivity that may be non-self-reactive, are present in human insulitic lesions. Establishing means and motive is a more nuanced venture, as their possible roles could be either pathogenic or protective, passive, or co-conspirators to the crime. Nevertheless, we will conclude that credible evidence exists for a role of non-islet-reactive T cells in disease.

## Investigating the Scene of the Crime: Evidence of Criminal Action Within Islets in T1D

### The Victims: Pancreatic Islet β Cells

In T1D, a scenario in which the scene of the crime is the pancreatic islet and the victim is the β cell can be contemplated. Arriving on this scene, our investigator is confronted by a chilling scene: a small crowd of infiltrating cells has gathered (first termed insulitis in 1902 by Schmidt (6), observing the pancreas of a 10-year-old child with diabetes). These infiltrates are diverse in location and composition. Insulitis is present in a small percentage of islets (even at diagnosis), making it easy to miss when examining small numbers of islets. The nature of β-cell destruction seems sporadic, almost random. Analysis of pancreas biopsies from adult living individuals taken only weeks from diagnosis (7) showed that insulitis [defined as ≥15 CD45^+^ cells/islet (8)] affected only 11% of the islets at diagnosis. At onset, insulitis is present in 25.8% of the insulin containing islets (ICIs) versus 2.9% of the insulin deficient islets (IDIs) (8). Thus, these snapshots of evidence of insulitis are concentrated in islets with insulin expression, indicating focused aggression against functional β cells.

Factors in the islet microenvironment that could serve to provoke immune attack remain under study. As mentioned above, remodeling events (waves of apoptosis that prime the immune system: “remodeling gone wrong”) that occur early in life could set the stage for subsequent unlawful entry (5). Once the stage has been set, islet α and β cells in T1D could express the CXCL10 chemokine. CXCR3 expression in T cells has been identified in inflamed pancreata and pancreatic draining lymph nodes from donors with T1D (9, 10) along with islet β-cell expression of monocyte chemoattractant protein-1 [MCP-1 (11)]. Further potential contributors to a chemoattractive/pro-inflammatory environment are the expression of IFN-α (12), and later in disease progression, IL-1β (13), the latter potentially secreted by virally infected β cells or by α cells (14). In addition, the scene in the islets appears altered with increased HLA Class I (HLA-I) expression in both α and β cells (15) and changes in the extracellular matrix (16) (among others) that can be interpreted as signs of a confrontation.

### Gathering Evidence at the Scene of the Crime

A survey of human insulitis reveals a diverse array of suspects, composed of CD8^+^ and CD4^+^ T cells, B cells, macrophages, and other immune cells (17–22) [along with data sets from consortia such as the Network of Pancreatic Organ Donors with Diabetes (nPOD; https://www.jdrfnpod.org/), the Exeter Archival Diabetes Biobank (EADB; https://foulis.vub.ac.be/) and the Human Islet Research Network (HIRN; https://hirnetwork.org/)]. Islet-reactive T cells emerge as prime suspects of β cell killing. These have been unambiguously identified in the pancreas using HLA-I multimers (MMrs) (23–27), and implicated through *in vitro* assays using HLA class II restricted peptides with many specificities defined in peripheral blood which have been confirmed among islet infiltrating T cells (28–30). Insulitic CD8^+^ T cells are present in higher numbers than other immune cell types. They increase in number as insulin decreases and decline once insulin positive cells can no longer be detected (21). This suggests a major role, but did these prime suspects act alone or with assistance from co-conspirators? This surge in T cell numbers represents an ‘opportunity’ for non-islet reactive T cells to also enter the islets (in response to the chemoattractive and pro-inflammatory factors), interact with antigen presenting cells (APCs) and reinforce islet-reactive T cell responses.

Other immune cell populations follow a similar trend, albeit in lower numbers. These include CD4^+^ T cells with only a few detected in human islets. Their numbers are slightly increased in the pancreas at the autoantibody positive stage, along with an increase in CD11c^+^ cells (31). This could indicate that CD4^+^ cells and APCs play a role in disease initiation, which would be consistent with Non Obese Diabetic (NOD) mouse studies (32) and a recent single cell analysis that demonstrated a progressive increase of intra-islet leukocyte infiltration and significant heterogeneity in cell type and function (33). CD20^+^ B cells, macrophages and other immune cells are also present in insulitis or in peri-insulitis. Their presence and numbers are associated with length of disease and age at disease diagnosis (18–20, 34–37). Only a few studies have attempted to identify CD4^+^ regulatory T cells (Tregs) in the human pancreas with some success (38), but other studies did not confirm the presence of conventional CD4^+^ Tregs in human insulitis (18, 20). It remains to be demonstrated (with better tools) whether regulatory cells are overwhelmed by the developing effector response, incapable of providing adequate T cell regulation, or if they are absent altogether. Such dereliction of duty by immune regulation/Tregs would be a notable contributing factor to the crime that took place.

### Arraigning the Key Perpetrators/Prime Suspects: Islet Reactive T Cells in Human T1D

Although the number of antigens reported to be recognized by insulitic CD4^+^ and CD8^+^ T cells from donors with T1D is limited, CD8^+^ epitopes from native and neoepitopes from islet-derived proteins have been identified (25, 26, 39) along with broad CD4^+^ T cell reactivity (28–30); see (40) for a review of islet proteins with native and neoantigen epitope targets. Two reports documented higher numbers of pancreas-infiltrating cells reactive to zinc transporter 8 and epitopes derived from previously unidentified targets in donors with T1D than healthy controls (25, 26). Bender et al. demonstrated that preproinsulin reactive CD8^+^ T cells were enriched in the islets of donors with T1D. These cells preferably infiltrated ICI, with a mean frequency of 40% (of total CD8^+^ T) cells compared to 22% in IDIs. Preproinsulin-reactive CD8^+^ T cells were also present in the exocrine pancreas of non-diabetic donors, and their numbers increased in donors with islet autoantibodies and T1D (23). What is the role of T cells found in the pancreatic exocrine tissue in T1D? To date, this is unknow, but the identification of the reactivity and extended phenotype of these T cells, their possible reactivity to islet targets, or exocrine or duct components, will require investigation to understand their role in T1D. Rodriguez-Calvo et al. showed that 10% of CD8^+^ T cells react against preproinsulin in the pancreas of living individuals with T1D whose specimens were obtained only weeks from diagnosis (27). Although some of these CD8^+^ T cells express the memory marker CD45RO in human pancreas specimens (23, 26), the question of whether this memory phenotype is a universal feature and its precise nature remains unsettled. Islet-reactive CD8^+^ T cells have been shown to produce inflammatory and cytotoxic mediators (25, 26), indicating sufficient means to commit the crime. Thus, islet-reactive T cells have the means, motive, and opportunity to commit the crime of β cell damage.

## Investigating Alleged Co-Conspirators: Evidence of Criminal Action by Non-Islet-Reactive T Cells in T1D

Islet reactive T cells were arraigned as our prime suspects but did these suspects act alone or with assistance from accomplices? Here, we consider potential roles for non-islet-reactive T cells, including active involvement, indirect contributions that either promoted or potentiated the crime, and modestly heroic attempts to regulate the aggressiveness of the islet-reactive T cell attack on β cells. To build our case, we will draw on T cell data from animal models and from human T1D.

### Non-Islet-Reactive T Cells in Animal Models

Although animal models of T1D do not completely reflect the human disease, they are a crucial tool to perform real-time exploration of mechanisms of immune regulation in the pancreas and associated lymphoid organs. In the lymphocytic choriomeningitis virus-rat insulin promotor-glycoprotein (LCMV-RIP-GP) mouse model of T1D, as little as 1% of the islet T cell infiltrate consists of CD8^+^ T cells specific for the driver antigen in this monospecific model (41). In this transgenic (i.e. β cells transgenically expressing experimental driver antigens), non-islet-reactive CD8^+^ T cells with known specificity towards irrelevant antigens were transferred and activated *in vivo* during disease onset (42). Titration of the numbers of non-islet-reactive CD8^+^ T cells revealed that these cells halted T1D onset in a dose-dependent manner. High numbers of non-islet-reactive infiltrating CD8^+^ T cells left β cells largely unharmed despite islet-specific CD8^+^ T cells being also present at the site. Another study showed that inflammation induced by diabetogenic CD4+ T cells alone was not sufficient to mediate non-islet reactive CD4+ T cell islet entry and/or retention (43). In addition, islet antigen expression was a key factor in determining the ability of a given T cell population to accumulate in the islets (43). Some evidence suggests that non-islet-reactive T cells may have different entry requirements than islet-reactive T cells. For example, in one study, the entrance of non-islet-reactive T cells required a chemokine response and VCAM-1 expression by the islets (44).

In multiple sclerosis (MS) and the experimental autoimmune encephalomyelitis (EAE) mouse model of the disease, the Davis lab reported an opposing immune response against the autoimmune CD4^+^ T cells driving the disease (45). Clonal expansion of endogenous CD8^+^ T cells not reactive to the myelin driver antigen was found, and the target peptides were identified for two clones. Co-immunization of mice with the disease-driving antigen myelin oligodendrocyte glycoprotein and the newly discovered peptides protected mice from EAE through expansion of regulatory CD8+ T cells expressing CD122 and Ly49. These markers have been found to be expressed on regulatory CD8^+^ T cells in other studies (46).

It is evident from these model studies and others on islet-infiltrating T cells (and from those infiltrating most other inflammatory sites) that T-cell recruitment to islets (or other sites of inflammation) is antigen independent (e.g., viral reactive cells at autoimmune sites), with an antigen-dependent component (43), with the likelihood that both mechanisms rely on diverse interstitial chemokines and upregulation of integrins on the vascular endothelium (47, 48).

### Non-Islet-Reactive T Cells in Insulitis in Humans

Establishing an opportunity that would allow the crime to occur boils down to showing that the suspect was present at the scene of the crime. Non-islet-reactive T cells would be drawn to a pro-inflammatory and chemoattractive environment in islets: this establishes ‘opportunity’ for non-islet-reactive T cells to be present *in situ*. Although informative, the strategies [MMr staining (23–27), growth from islets (28, 30), sorted T cells for T cell receptor (TCR) transductant generation (29)] to identify the targets of autoreactive T cells in human insulitis have not yet defined the relative representation of islet specificities among the total number of islet-infiltrating CD4^+^ and/or CD8^+^ T cells. Moreover, these strategies are inherently biased by their focus on epitopes known to be targeted by circulating T cells, which could be different in the pancreas (26). The antigen specificities for a large fraction (96%) of TCR transductants generated from single T cells seen in (29) or T cell lines and clones derived from insulitis remain unassigned (23, 25–28). This raises two possibilities: 1) a significant proportion of islet infiltrating T cells recognize self-antigens that are not yet appreciated as being disease relevant or 2) a significant proportion of T cells found in the islets are not islet-reactive. Collectively, the relative representation of islet-infiltrating T cells recognizing islet *vs.* non-islet antigens and the hierarchy of the islet antigens recognized remain open questions.

### Interacting With the Crime in Progress: Indirect Roles for Non-Islet-Reactive T Cells in T1D

**Figure 1:**
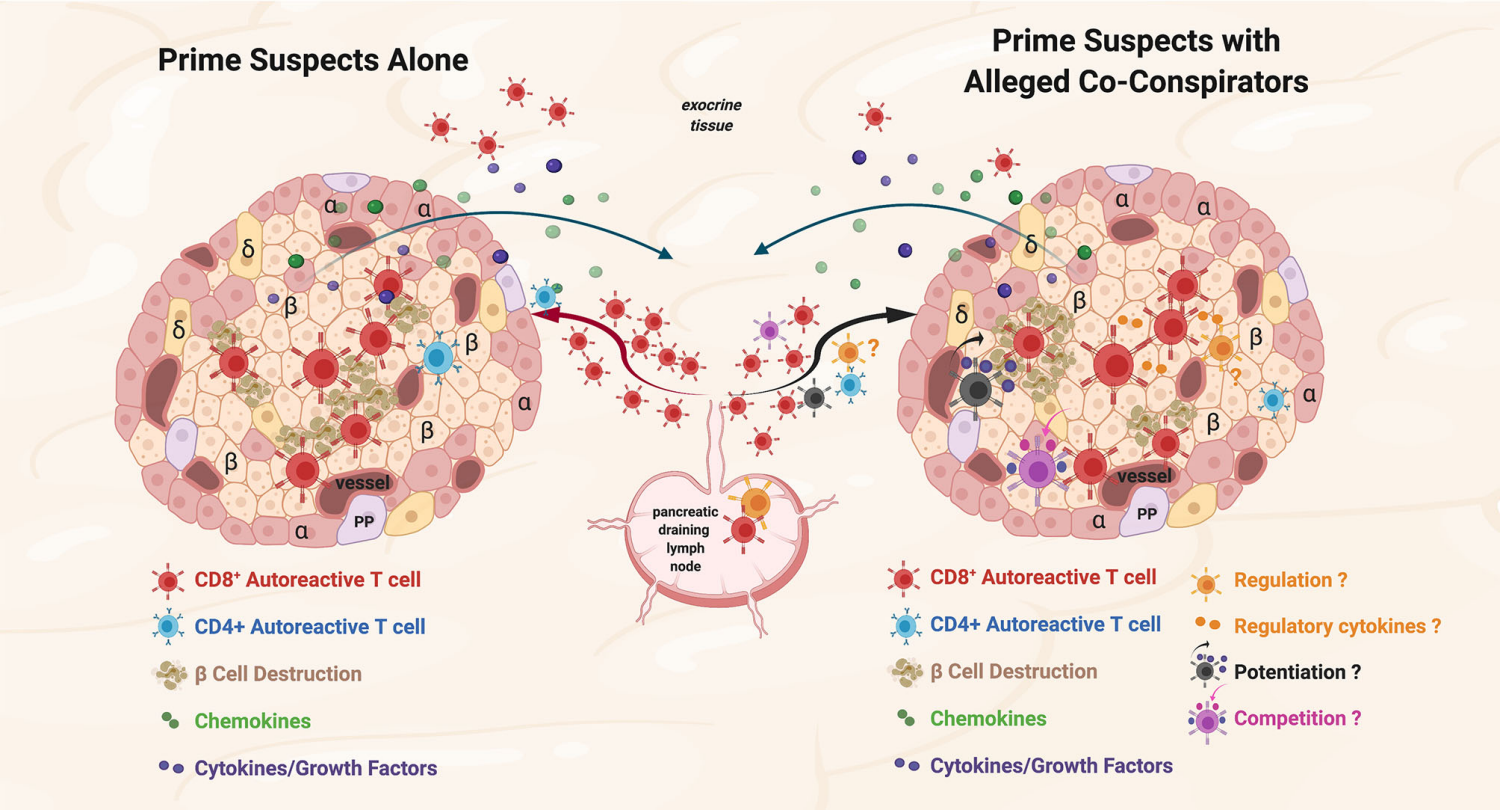
Prime suspects and alleged co-conspirators at the scene of the crime: function of islet-reactive T cells and potential function(s) of non-islet-reactive T cells in insulitis. Proposed schema for islet and non-islet cell reactive T cell functions in insulitis. Representative alpha (α), beta, (β) and delta (δ); cells, and blood vessels are labeled and shown in the represented islet on the left and on the right. The islets are depicted as sources of chemoattractants/cytokines gradients (depicted in green and purple symbols, respectively) through venules with the resultant egress of lymphocytes from the pancreatic draining lymph nodes (pLN) and migration to the islets. Autoreactive CD4^+^ T cells are depicted in blue. On the left, the prime suspects, β−cell cytolytic function of islet-reactive CD8^+^ T cells (depicted in brick red) with β cell destruction depicted in brown. For both representative islets, auto-reactive CD8^+^ T cells are shown in the exocrine tissue. On the right, three potential functions of the alleged co-conspirators, non-islet-reactive T cells, on β−cell destruction by the prime suspects, are depicted. A pro-inflammation ‘potentiating’ T cell (depicted in black) with cytokine/growth factor secretion (depicted in green and purple) and increased β−cell destruction are depicted in brown. The potential suppressive function of a Treg on β−cell destruction and the potential regulatory effects of cytokines are depicted in orange. The competitive function(s) of a resource/nutrient/growth factor consuming T cells are depicted. The three proposed functions of non-islet-reactive T cells in insulitis are not necessarily mutually exclusive.

Non-islet-reactive T cells are present at the scene of the crime in animal models of T1D, in human T1D, and in other human autoimmune diseases, but what are their possible roles in the disease process? We consider three: 1) active regulatory cell function or secretion of regulatory cytokines/factors, 2) potentiation of the pro-inflammatory environment, and 3) bystander consumption of cytokines/growth factors/nutrients *in situ* (potentially dampening autoreactive T cell function) (**Figure 1**).

#### Regulation of the Microenvironment

As well-meaning citizens, active Tregs or T cells with regulatory cytokine secretory, or ‘exhausted’ properties may suppress autoreactive T cells *in situ*. In NOD mice, the suppressive mechanism of infiltrating Tregs seems to be of a less specific nature. Islet-reactive CD8^+^ T cells in the pancreas of protected mice showed greatly reduced proliferation rates and lower amounts of islet-reactive cells present, with some signs of exhaustion (42). In EAE, on the other hand, a perforin-dependent mechanism of suppression was found (45). Tregs strongly impact T1D pathogenesis in mouse models, with a gradual decrease in the Treg:T conventional (Tconv) cell ratio in inflamed islets (49). Tang et al. suggested that the decline in pancreatic Tregs reflected apoptosis; a reduced influx of Treg *vs.* Tconv cells may also be possible. In human insulitis, the data point to an absence of classical CD4^+^FOXP3^+^ Tregs. Current data from animal models supports multiple regulatory mechanisms, but the common thread is that Treg activity is inadequate to prevent the crime.

Another mode of microenvironment regulation in diverse mouse model systems is the local secretion of IL-4. Expression of IL-4 in β cells under the control of the human insulin promoter abrogated development of T1D (50, 51), but conversely, increased antigen presentation within islets (52). The use of an IL-4-Ig fusion protein suppressed proinflammatory cytokine production, but augmented islet infiltration (53). Interestingly, expression of CD1d, the ligand for NKT cells, under the control of the insulin promoter in β cells in NOD mice was associated with an IL-4-secreting phenotype of NKT cells and abrogation of T1D development (54). Collectively, these data in murine models suggest that skewing of the microenvironment with regulatory cytokines can have a beneficial effect for the development of T1D, but requires its own regulation.

#### Potentiation of the Proinflammatory Microenvironment

Non-islet-reactive T cells may be propagators of violence (“egging on the perpetrators”) by secretion of pro-inflammatory cytokines (e.g. IFN-*γ*) or IL-2, which can potentiate and support the replication and function of CD8^+^ autoreactive T cells. IL-6 has an unclear role in the development of T1D as the expression of IL-6 from both α and β cells was found to be reduced in islets of donors with T1D as compared to those of control donors (55). In mouse models, overexpression of IL-6 has been found to be insufficient to induce T1D, but rather increased islet infiltration by predominantly B cells, but also CD4^+^ T cells and macrophages (56) whereas IL-6 inhibition has been found to reduce incidence (57). Proinflammatory cytokines, possibly secreted by bystander T cells, are known to contribute to β cell stress (58–60) and to directly damage β cells (61–63).

A potential source of bystander T cell activation in the pancreas is viral infection. Bystander activated CD8^+^ T cells have been investigated in NOD mice infected with Coxsackievirus B3 and diabetes acceleration was observed. However, this required a pre-existing level of insulitis at the time of infection (62, 63). Of note, viral infections have also been found to be protective in T1D models (64), suggesting that the specific influence from non-islet reactive T cells may be largely dependent on situation and timing. In different models, bystander activated CD8^+^ T cells have been implicated as either benefiting or harming the microenvironment (65), but the strongest evidence seems to suggest a harmful role.

#### Depletion of Nutrients and Growth Factors

Non-islet-reactive T cells could contribute to the crime scene by “taking up space”. The tumor microenvironment is highly metabolically active to support tumor growth. This leads to a rapid depletion of nutrients in tumors with high glycolytic rates, and the microenvironment becomes metabolically restrictive for infiltrating T cells leading to resistance to T cell cytotoxicity (64). Could similar mechanisms be at play in an islet infiltrate consisting of a large amount of non-islet-reactive cells? Competition over nutrients can occur between immune cells. One example is the interaction between T cells and APCs, where APCs can become nutrient-starved by clusters of neighboring activating CD8^+^ T cells (65). Similar clustering was observed in mouse models of suppression (42). Depletion of arginine and tryptophan in the microenvironment may also shift the inflammatory response. Arginine is utilized for nitric oxide (NO) production in inflammatory macrophages, and is important for T cell responses (66). Tryptophan is depleted by the enzyme indoleamine 2,3-dioxygenase (IDO), and the expression of IDO, for example, in tolerogenic APCs leads to inhibition of T cells through both lack of tryptophan and the presence of the resulting metabolite kynurenine (67). T cells increase their metabolic demand upon stimulation and activation. Consequently, depletion of nutrients is a potential regulatory factor worthy of further exploration in the context of insulitis. Competition for nutrients and growth factors by non-islet reactive T cells in insulitis could contribute to the indolent nature of T1D development in humans.

## Evidence of Criminal Action by Non-Target Reactive T Cells in Other Autoimmune Diseases and in Viral Infections: Is There A Common Mode Of Action?

In rheumatoid arthritis (RA), epidemiologic and clinical observations suggest that Th1 responses are primary drivers of disease (68). This is illustrated by the amelioration of RA in pregnant women, who present improved symptoms and diminished inflammation driven by a shift from Th1 to Th2 T cell responses (69). However, given that relapses occur six months after delivery in most cases, the beneficial Th2 response is not maintained long term. These observations have implications for disease pathogenesis, as they indicate that T cell responses (antigen-specific and bystander) have plasticity and can be effectively modulated. If modulation could be maintained, T cells might no longer find a motive to go to the target tissues or might have more limited means to cause injury. In RA, a disease setting in which sampling disease-proximal joint tissues is feasible, MMr staining of T cells derived from the synovial fluid revealed a notable population of influenza-reactive CD4^+^ T cells (70). This indicates that non-tissue specific T cells are drawn to sites of inflammation. These T cells displayed a memory phenotype, but were CD25-negative, suggesting that they had not been recently activated.

MS has been linked to EBV infection and, although up to 95% of the general population is seropositive, disease risk is 15-fold greater in seropositive compared to seronegative individuals (71). Patients show increased T cell responses against EBV nuclear antigen (EBNA1), which cross-recognize myelin antigens, whereas influenza and CMV-specific responses are not altered (72). Analogously, *in situ* MMr staining showed that 5% of CD8^+^ T cells in the pancreas of 4 recent onset T1D subjects were reactive against CMV (27). These cells were located predominantly in the exocrine tissue, whereas their presence around or inside the islets was rare. Likewise, a recent study identified T cells that could be activated either by influenza or GAD65 (73), but these cross-reactive cells were from peripheral blood and not documented within the islets.

The role of virus-reactive T cells in the pathogenesis of human autoimmune diseases remains unclear. Given the potential cyclical activation of T cells due to new infections or viral reactivation, virus-reactive T cells might be important contributors to tissue inflammation. T1D has been historically associated with enterovirus infections (74). Investigating the location and phenotype of enterovirus-reactive cells has potentially important implications for disease pathogenesis and prevention. Although molecular mimicry between microbial and β-cell epitopes has been described, the arrival of virus-reactive T cells could be driven by viral antigens and/or chemokines/cytokines, with the aim of eliminating infected β cells. Their ongoing activity could contribute to islet inflammation by activating anti-viral responses (interferon-mediated), favoring antigen presentation (increasing HLA-I expression), and inducing β cell destruction. With ~96% of the reactivities of islet infiltrating T cells undefined, the question remains open as to the identity and *in situ* function of this large portion of T cells.

## Conclusions and Future Perspectives

Collectively, there is some consensus that in T1D (and other autoimmune diseases), T cells that lack any documented self-reactivity are present within inflamed tissues. Some of these might recognize unknown self-antigens, but others have been shown to recognize epitopes from common viruses. It cannot be completely ruled out that non-islet reactive infiltrating T cells could actively suppress autoreactive T cells or restrain their activity by depleting nutrients and growth factors/regulatory cytokines or competing for ‘space’. While we cannot exclude that multiple mechanisms may be at play, the most likely conclusion is that non-islet reactive T cells potentiate and support the replication and function of destructive autoreactive T cells by secreting pro-inflammatory cytokines and growth factors. It should be noted that nutrient consumption and cytokine secretion within the islets could also have a direct negative effect on β cells, leading to a direct, but non-antigen specific contribution to autoimmunity in T1D. Although the current evidence is inadequate to convict, we assert that further investigative efforts to scrutinize the motive and opportunity of non-specific islet infiltrating T cells is warranted. Analogous to conducting interviews, constructing a psychological profile, and petitioning the court for a search warrant, investigators should assess the function of non-islet-reactive T cells, their activation state, their ability to secrete regulatory, pro-inflammatory or growth factors, their interactions with other immune cell types, their cell surface phenotype, TCR repertoire, and their function in animal models. These studies should be paired with companion analysis of the ‘scene of the crime’ in T1D to confidently determine the complicity and culpability of these T cells so that they can be brought to justice.

## Author Contributions

All authors actively contributed to writing and revising the article and approved the submitted version. All authors are accountable for the content of the work.

## Funding

GC is supported by grants from the Swedish Research Council, the Swedish Society for Medical Research, the Göran Gustafsson Foundation, and the Science for Life Laboratory. EJ is supported by the National Institute of Diabetes and Digestive and Kidney Diseases (Human Islet Research Network U24 DK104162). SK is supported by the National Institute of Diabetes and Digestive and Kidney Diseases (UC4 DK116284), and SK is the George F. and Sybil H. Fuller Term Chair in Diabetes. RM is funded by grants from The Leona M. and Harry B. Helmsley Charitable Trust (Helmsley no. 1901-03689), the Fondation pour la Recherche Médicale (EQU20193007831) and the Agence Nationale de la Recherche (ANR-19-CE15-0014-01). RM and TR-C are funded by the Innovative Medicines Initiative 2 Joint Undertaking under grant agreements 115797 and 945268 (INNODIA and INNODIA HARVEST). These Joint Undertakings receive support from the Union’s Horizon 2020 research and innovation programme, European Federation of Pharmaceutical Industries Associations, JDRF and The Leona M. and Harry B. Helmsley Charitable Trust. MH is supported by NIH R01 AI134971 and NIH R01 AI092453.

Research in the laboratories of authors (SK, RM, TR-C, MH) is performed with the support of the Network for Pancreatic Organ donors with Diabetes (nPOD; RRID : SCR_014641), a collaborative type 1 diabetes research project supported by JDRF (nPOD: 5-SRA-2018-557-Q-R) and The Leona M. & Harry B. Helmsley Charitable Trust (Grant#2018PG-T1D053). Organ Procurement Organizations (OPO) partnering with nPOD to provide research resources are listed at http://www.jdrfnpod.org/for-partners/npod-partners/.

## Disclaimer

The content and views expressed are the responsibility of the authors and do not necessarily reflect the official view of nPOD.

## Conflict of Interest

MH is an employee of Novonordisk; no Novonordisk drugs are discussed in this article.

The remaining authors declare that the research was conducted in the absence of any commercial or financial relationships that could be construed as a potential conflict of interest.
